# The Effects of Antidepressants “Fluoxetine and Imipramine” on Vascular Abnormalities and Toll Like Receptor-4 Expression in Diabetic and Non-Diabetic Rats Exposed to Chronic Stress

**DOI:** 10.1371/journal.pone.0120559

**Published:** 2015-03-31

**Authors:** Mohamed Habib, Safaa Shaker, Nesreen El-Gayar, Sawsan Aboul-Fotouh

**Affiliations:** 1 Department of Pharmacology, Faculty of Medicine, Ain Shams University, Cairo, Egypt; 2 Department of Histology, Faculty of Medicine, Ain Shams University, Cairo, Egypt; 3 Clinical Pharmacology Unit, Faculty of Medicine, Ain Shams University, Cairo, Egypt; University of São Paulo, BRAZIL

## Abstract

Several studies reveal that diabetes doubles the odds of comorbid depression with evidence of a pro-inflammatory state underlying its vascular complications. Indeed, little information is available about vascular effects of antidepressant drugs in diabetes. Method: We investigated the effect of chronic administration of fluoxetine “FLU” and imipramine “IMIP” on behavioral, metabolic and vascular abnormalities in diabetic and non-diabetic rats exposed to chronic restraint stress (CRS). Results: Both diabetes and CRS induced depressive-like behavior which was more prominent in diabetic/depressed rats; this was reversed by chronic treatment with FLU and IMIP in a comparable manner. Diabetic and non-diabetic rats exposed to CRS exhibited abnormalities in glucose homeostasis, lipid profile and vascular function, manifested by decreased endothelium-dependent relaxation, increased systolic blood pressure and histopathological atherosclerotic changes. Vascular and metabolic dysfunctions were associated with significant increase in aortic expression of TLR-4, and pro-inflammatory cytokines (TNF-α and IL-1ß). FLU ameliorated these metabolic, vascular and inflammatory abnormalities, while IMIP induced either no change or even worsening of some parameters. Conclusion: FLU has favorable effect over IMIP on metabolic, vascular and inflammatory aberrations associated with DM and CRS in Wistar rats, clarifying the preference of FLU over IMIP in management of comorbid depression in diabetic subjects.

## Introduction

The concurrence of depression and diabetes mellitus (DM) constitutes a major health problem. Among people with DM, whose risk of depression is 50–100% greater than the general population [1], depression is associated with higher complication [2] and mortality [3] rates. Moreover, depression may impair glycemic control, treatment compliance and increase the risk of vascular complications in DM [4]. Additionally, the response to antidepressants has been reported to be altered in diabetics [5]. Treatment with antidepressants has also been reported to affect glucose homeostasis in diabetic individuals. Although DM risk is elevated for the major antidepressant classes, the risks posed by individual medications may vary widely [6]. Several studies indicated that the effect of selective serotonin reuptake inhibitors (SSRIs) on glycemic control is quite controversial. Paroxetine and fluvoxamine has been reported to carry an increased DM risk while elevated risk was not associated with fluoxetine, citalopram, or sertraline [7]. In contrast, a hypoglycemic effect was reported with fluoxetine or paroxetine while other studies failed to confirm this finding and still others noted a worsening of glycemic control [8]. Indeed, little information is available about vascular effects of antidepressant drugs in DM.

Vascular disease is the most important complication underlying most of the disabilities and deaths in diabetic patients. Endothelial dysfunction is a cardinal feature of both types of DM, and is believed to be involved in the pathophysiology of diabetic vasculopathy [9]. Additionally; persistent depressed mood substantially increases the risk of cardiovascular disease via formation of atherosclerosis which represents a series of metabolic changes and cellular inflammatory responses [10]. Adipokines, such as tumor necrosis factor-α (TNF-α), interleukin-1β (IL-1β), and interleukin-6 are associated with development of insulin resistance and vascular disease [11].

Evidence is accumulating that TLR4 plays an important role in the pathogenesis of atherosclerosis [12]. The expression of TLR-4 has been detected in various types of cells including T cells, monocytes, macrophages, and dendritic cells. Activation of TLR-4 results in NF-κB activation and subsequent induction of vascular pro-inflammatory cytokines e.g. TNF-α and IL-1β that have also been demonstrated in inflammation after sub-acute and chronic stress and insulin resistance [13, 14]. Therefore, there is a plausible linkage of TLR4 to the production of pro-inflammatory cytokines, which, in turn, contribute to vascular dysfunction associated with depression and DM.

The present study was designed to investigate the effect of chronic treatment with a SSRI (fluoxetine) and a tri-cyclic antidepressant (imipramine) on depressive-like behavior, endothelial dysfunction, glucose homeostasis, lipid profile, and vascular expression of TLR4, TNF-α and IL-1β in diabetic and non-diabetic rats exposed to chronic restraint stress, experimental model of depression, in a trial to justify the preference of one antidepressant agent over the other for management of comorbid depression in diabetic subjects.

## Materials and Methods

### Animals

Male Wistar rats (170–200 g) were purchased from National Research Centre (Dokki, Giza, Egypt.) and left to acclimatize for one week prior to beginning of the experiment. Rats were kept in constant environmental conditions; temperature≈25°C, relative humidity 50–60% and 12 h light/dark cycle (lights on at 6 am and off at 6 pm). All experimental procedures were performed in accordance with The European Community guidelines for the use of experimental animals [15]. The present study was approved by the Research Ethics Committee of Faculty of Medicine, Ain Shams University (FMASU-REC). FMASU-REC operates under Fedral Wide Assurance. The Number of approval is FWA 00006444.

### Experimental Models

#### Model of type II diabetes

To obtain insulin resistant type II diabetes, rats were fed high-fat diet ‘HFD’ as described by Aboul-Fotouh and Elgayar [16], for a period of 2 weeks followed by a single intraperitoneal (i.p.) injection with 35 mg/kg streptozotocin ‘STZ’. Rats received 5% sucrose solution orally for the first 48 h after STZ injection to minimize death from hypoglycemia. On the 3^rd^ day, tail blood samples were obtained by a sharp cut and blood glucose concentration was measured using gluco-check apparatus (Accu-Check Active, Germany). Rats with blood glucose levels above 200 mg/dl were considered diabetic. Streptozotocin (Sigma-Aldrich chemicals Co., Germany) was dissolved in 0.1 M sodium citrate buffer (PH = 4.4).

#### Chronic Restraint Stress (CRS)

Rats were placed in Plexiglas restrainers (25 cm × 8 cm) for 4 h (9:00 am to 1:00 pm) per day during 6 weeks. The restrainer was wide enough to allow comfortable breathing but restricting rat's movement with air vents at the nasal end. The stress procedure was carried out in a different room [17, 18]. Diabetic rats began to be exposed to CRS 3 days after STZ injection.

### Treatments and Experimental Groups

Imipramine hydrochloride and fluoxetine hydrochloride powders (Sigma-Aldrich chemicals Co., Germany) were dissolved in saline and administered i.p. in a volume of 2 ml/kg. Ninety-two rats were divided into 2 main groups. The first group (non-diabetic rats) was subdivided into 4 subgroups: Naïve group (n = 10); not exposed to CRS, CRS vehicle-treated group (n = 11); exposed to CRS for 6 weeks and received vehicle i.p., CRS FLU-treated group (n = 14); received fluoxetine 10mg/Kg/day i.p. [19] and CRS IMIP-treated group (n = 14); received imipramine 10mg/Kg/day i.p [20]. The second group (diabetic rats) fed HFD for 2 weeks followed by STZ 35 mg/kg, i.p) and was subdivided into 4 subgroups: Control DM group (n = 10); not exposed to CRS, DM/CRS vehicle-treated group (n = 9); exposed to CRS for 6 weeks and received vehicle i.p., DM/CRS FLU-treated group (n = 12); received fluoxetine and DM/CRS IMIP-treated group (n = 14); received imipramine. All treatments were received following the daily stress regimen for the last 3 weeks of CRS.

### In-vivo Studies

#### Behavioral tests. Open Field Test (OFT)

OFT was used to detect spontaneous locomotion and anxiety-related behaviors in rats. Rats were placed in the center of a quadrangular arena (60 x 60 cm) divided into 16 equal squares and the number of crossed squares (visited with all four feet on one square) during 5 minutes was recorded [21].

#### Forced swimming test (FST)

In FST, rats were forced to swim in a vertical glass cylinder (diameter 22.5 cm, height 50 cm) containing 35 cm of fresh water at ≈ 25°C. After open field test, the rats were forced to swim for 15 min and thereafter dried with towel and returned to their cages. After 24 h, rats were re-exposed to the forced swimming for 5 min and behavior was videotaped and immobility time (during which the animal floated on the surface with its front paws together making only those movements necessary to keep it afloat) was analyzed. Depressive-like behavior was inferred from “despair” as indicated by increased duration of immobility [21].

#### Measuring body weight and systolic blood pressure

Body weights of animals were recorded at the beginning of the study and weekly thereafter. Systolic blood pressure was measured using indirect tail cuff plethysmography with an inflatable cuff and a pulse sensor placed around the tail coupled to a ML 125 NIPB controller connected to the plethysmograph (ADI instrument, Australia). The inflated cuff pressure was computed using power lab/85p (ML 785 software program). SBP was calculated as the mean of 3 readings.

#### Insulin tolerance test (ITT)

At the end of the experiment, insulin (0.75 IU/kg, i.p.) was administered and blood glucose concentrations were measured at 0, 30, 60, 90 and 120 minutes by using gluco-check apparatus. The value was presented as a percentage of initial blood glucose level [16].

### Ex-vivo Studies

#### Biochemical and molecular study. Assessment of glucose homeostasis and lipid profile

At the end of the study rats were anesthetized with urethane (1.2 gm/Kg, i.p.). Blood samples were collected in test tubes through retro-orbital approach and centrifuged at 3000 rpm for 15 min to obtain serum for biochemical estimation Total cholesterol (TC), triglyceride (TG), high-density lipoprotein–cholesterol (HDL-C), low density lipoprotein–cholesterol (LDL-C), and fasting blood glucose (FBG) levels were determined using commercially available enzymatic assays. Fasting blood insulin (FBI) level was measured by insulin Rat-ELISA kit (ALPCO Diagnostics- Catalog No. 80-INSRT-E10). Insulin sensitivity was estimated using the homeostasis model assessment of insulin resistance (HOMA-IR) as follows: FBG (mmol/L) X FBI (mIU/L)/22.5. HOMA-IR ≥2.8 represents insulin resistance state [22].

#### Determination of serum corticosterone

Serum corticosterone was measured by rat corticosterone ELISA kit (DRG international, USA; Cat. No. EIA-5186) according to the manufacturer’s instructions. Absorbance was measured at 450 nm and the lowest analytical detectable level of corticosterone was 4.1 ng/mL

#### Assessment of TNF-α, IL-1ß and TLR-4 proteins in aortic tissue homogenate

TNF-α, IL-1β and TLR-4 proteins were determined in aortic tissue homogenate using commercially available rat TNF-α ELISA kit (Quantikine, USA), rat IL-1β ELISA kit (Kamiya biomedical, USA) and rat TLR-4 ELISA kit (MyBioSource, USA) according to the manufacturer’s instructions. Absorbance was measured at 450 nm and the lower limit of detection for TNF-α and IL-1β kit was 12.5 pg/ml, 15.6 pg/ml and 0.625 ng/ml respectively. The protein content of aortic homogenate was determined using the method described by Bradford [23].

#### Estimation of TLR-4 gene expression by semi-quantitative reverse transcriptase polymerase chain reaction (RT-PCR) technique in the aortic tissue

Total RNA was extracted from the aortae of different groups using TriFast in combination with inhibitor of RNase activity (PEQLAB Biotchnologie, GmbH, Germany) according to the manufacturer's protocol. cDNA was synthesized using reaction mix (GoTaq Green Master Mix (Promega, USA). Specific PCR primers (Metabion international AG, Germany) were used in RT-PCR; TLR-4 sense primer 5′- AGTTGGCTCTGCCAAGTCTCAGAT- 3′ and antisense 3′-TGGCACTCATCAGGATGACACCAT-5′ [24]. Primers for β-actin were used according to Caruso et al. [25]; sense primer 5´-ACCACAGCTGAGAGGGAAATCG–3´ and antisense Primer 3´-AGAGGTCTTTACGGATGTCAACG-5´. PCR product sizes for ß actin was found at 180 bp, TLR-4 product size was found at 299 bp. The PCR products were resolved on a 2% agarose gel and visualized by ethidium bromide staining. The staining intensity was evaluated using the Molecular Analyst software (Gel-pro 3.1, USA). The gel was removed from the electrophoresis apparatus and visualized using UV trans-illuminator. The gel was subsequently visualized and photographed by the Gel Documentation System (Gel Doc EQ, BioRad laboratories, USA). Results were expressed as relative densitometric units of TLR-4 gene expression in percentage (%), normalized to the values of β-actin mRNA used as an internal control. Semi-quantitation was done using “Quantity One” computer program software version 4.6.3, (BioRad laboratories, USA).

### Functional assessment of isolated thoracic aortas

After taking blood samples, the chest was opened and the thoracic aorta was rapidly and carefully dissected and placed into Krebs-Henseleit solution. Aortic rings (4–5 mm width) were prepared and mounted between stainless steel triangles in organ bath filled with oxygenated (95% O2 and 5% CO2) Krebs-Henseleit buffer (37 ° C, pH = 7.4) and left to equilibrate for 1–1.5 h. Isometric responses were measured with a force transducer (K30, Hugo Sacks Electronics, Freiburg, Germany) connected to a bridge coupler type 570 and the trace was displayed on a two-channel recorder (Lineacorder, HSE, WR 3310). Cumulative dose-response curve was constructed by cumulative addition of phenylephrine (32.7 pM- 1mM) to the bath and EC50 and E max were determined for each curve to assess vascular reactivity. After reaching the plateau of the phenylephrine–induced sub-maximal contraction (100μM), the rings were relaxed by exposure to a stepwise increase in acetylcholine (ACh) concentration (366 pM—3.66 mM) to assess endothelium-dependent relaxation. Sodium nitroprusside-induced relaxation (Endothelium-independent relaxation) was also assessed using increasing concentrations of sodium nitroprusside (1nM-10μM). The percentage relaxation from a sub-maximal phenylephrine-induced contraction was determined and computed for all groups.

### Histopathological and immunohistochemical study

Sections from the thoracic aorta were cut and fixed in 10% formalin. Five μm sections were stained by H&E. The width of the intima and media were measured by a colour image analyzer (Video Pro 32; Leading Edge Pty Ltd) as an early index of atherosclerosis. Intima /media ratio (IMR) = width of intima at maximum intimal thickness/width of media at maximum intimal thickness [26]. For immunehistochemical staining of TNF-α in aortic tissue, 4 μm sections were cut from the aorta blocks and mounted on charged slides for standard immunoperoxidase staining technique according to Hsu and Raine [27] using primary TNF-α monoclonal mouse antihuman antibody (J1D9; Thermo Fisher Scientific, Waltham, MA, USA) diluted to 1:100 and biotinylated secondary antibody anti-mouse IgG (Zymed lab,San Fransisco,CA). Optical density of TNF-α immune-staining was measured using the image analyzer (Leica Q 500 M C program).

### Statistical Analysis

The results were expressed as mean±SEM and statistical analysis was performed using computer program SPSS, version 17.0 (SPSS, Chicago, IL, USA). Data were analyzed by two-way analysis of variance (ANOVA) followed by Bonferroni's test post-hoc for inter-group comparisons. Repeated measure ANOVA test was utilized for insulin tolerance test to determine the effect of treatment, diabetes and time factors. Pearson’s correlation coefficient was used to assess the correlation between endothelium-dependent relaxation and the expression of inflammatory markers (TLR-4, TNF-α and IL-1β) in aortic tissue. Differences were considered statistically significant at P<0.05.

## Results

### Effect of tested drugs on behavioral changes in FST and OFT

**Fig 1:**
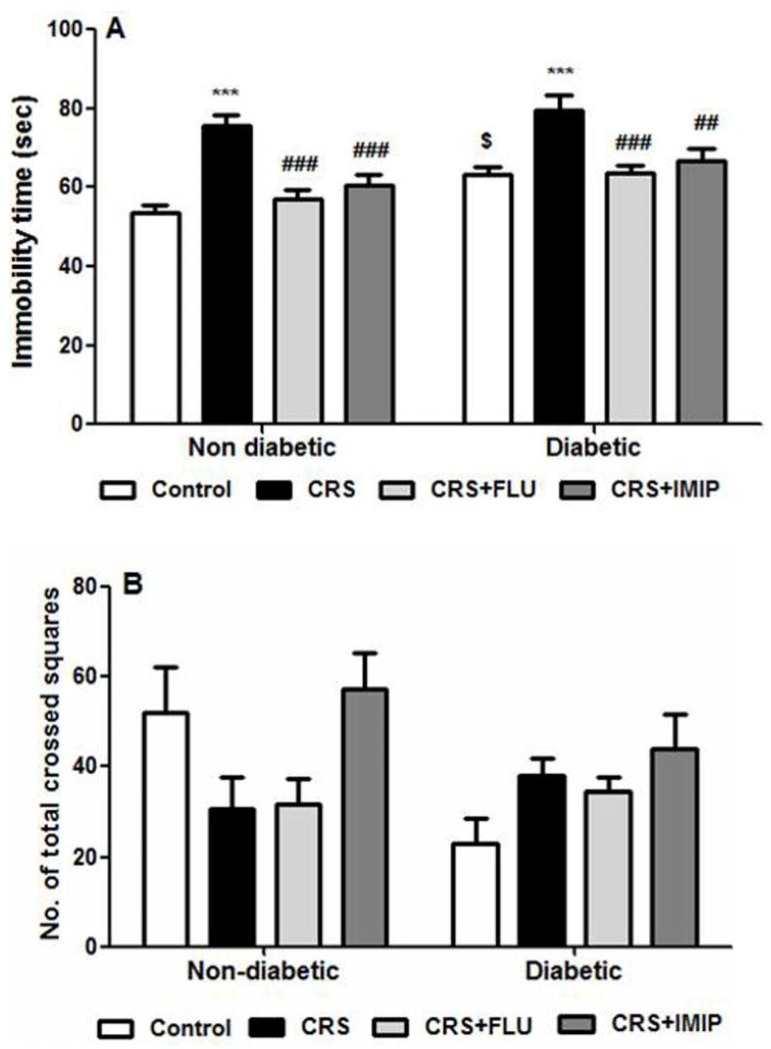
Effects of fluoxetine (FLU) and imipramine (IMIP) on forced swimming (A) and open field (B) tests behavioral changes in diabetic and non-diabetic rats exposed to chronic restraint stress (CRS). Data are mean±SEM of 9–14 rats/group. ***P<0.001 vs control group, ^##^P<0.01, ^###^P<0.001 vs. CRS group; ^$^P<0.05 diabetic vs. non-diabetic control rats by Two-way ANOVA with Bonferroni's post-hoc test.

Fig 1A reveals that induction of type 2 diabetes produced depressive-like symptoms in Wistar rats indicated by significant increase of immobility time in FST (F_(1,84)_ = 12.14, P = 0.0008). Exposure to chronic restraint stress (CRS) significantly (p<0.001) increased immobility time in both diabetic and non-diabetic rats compared to control groups. Two-way ANOVA revealed that chronic treatment with antidepressants, FLU and IMIP, significantly reversed CRS-induced changes in FST immobility time (F_(3, 84)_ = 19.27, P<0.0001) in both diabetic and non-diabetic rats compared to vehicle-treated groups. There was no significant difference between FLU and IMIP. At the same time, Fig 1B shows that the number of total crossed squares in OFT was decreased in non-diabetic and increased in diabetic rats. However, this effect was statistically insignificant indicating that rats’ locomotor activity was insignificantly affected by models and tested drugs.

### Effect of tested drugs on body weight

**Fig 2:**
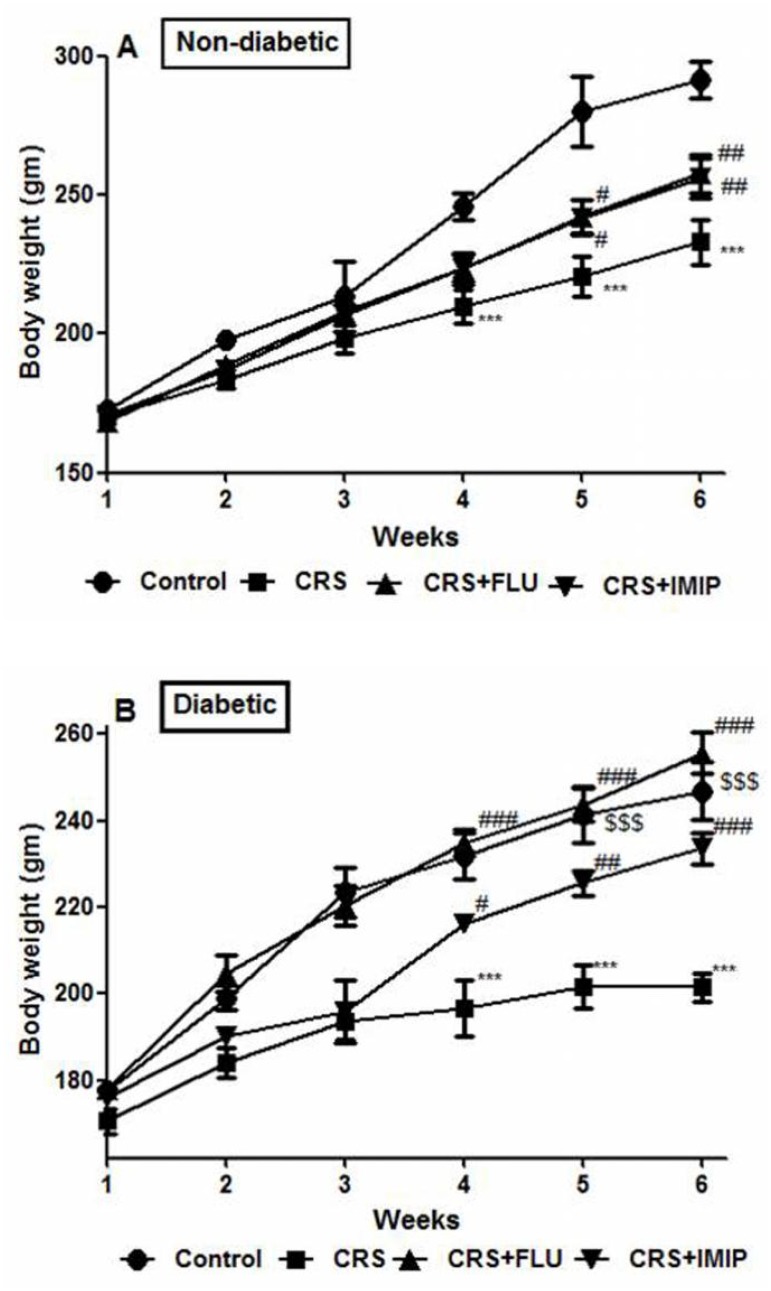
Effects of fluoxetine (FLU) versus imipramine (IMIP) on body weight in non-diabetic (A) and diabetic (B) rats exposed to chronic restraint stress (CRS). Data are mean±SEM of 9–14 animals per group **P<0.01,***P<0.001 vs. control group; ^#^P<0.05, ^##^P<0.01, #^##^P<0.001 vs. CRS group by Two-way ANOVA with Bonferroni's post-hoc test. ^$$$^P<0.001 diabetic vs. non-diabetic control rats.

As depicted in Fig 2, diabetic rats showed significant (P<0.001) reduction in body weight gain at the end of the 5^th^ and 6^th^ weeks. Exposure to CRS induced significant decrease in body weight gain at the end of the 4^th^, 5th and 6^th^ week in both non diabetic and diabetic rats (P<0.001) in contrast to control groups. Chronic treatment with fluoxetine and imipramine significantly reversed CRS-induced reduction of body weight gain at the end of the 5^th^ (P<0.05) and 6^th^ week (P<0.01) compared to vehicle-treated group. Repeated measures ANOVA revealed significant effect of time factor (F_(5,340)_ = 470.382, P = 0.000), time with treatment (F_(10, 340)_ = 12.216, P<0.0001) and time with diabetes (F_(5, 340)_ = 17.342, P <0.0001) interactions.

### Effect of tested drugs on systolic blood pressure (SBP)

**Fig 3:**
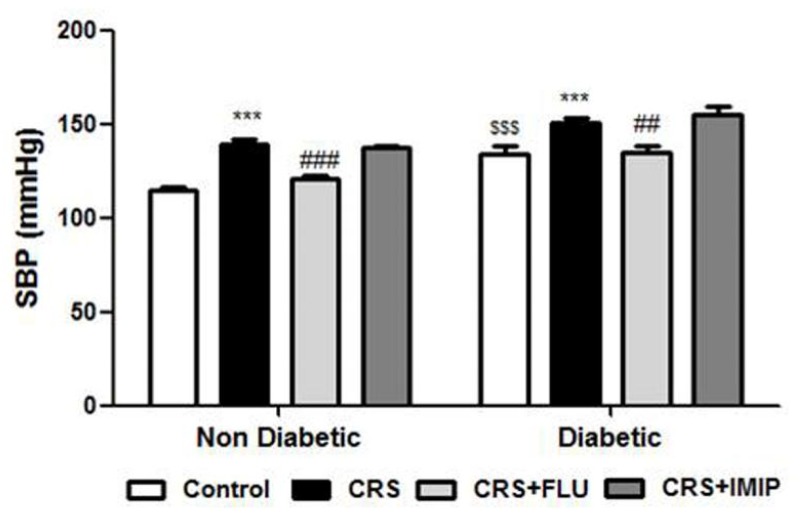
Effects of fluoxetine (FLU) versus imipramine (IMIP) on systolic blood pressure (SBP) in diabetic and non-diabetic rats exposed to chronic restraint stress (CRS). Data are mean±SEM (n = 6). ***P<0.001 vs. control group; ^##^P<0.01, ^###^P<0.001 vs. CRS group, ^$$$^P<0.001 diabetic vs. non-diabetic control rats by Two-way ANOVA with Bonferroni's post-hoc test.

As shown in Fig 3, diabetic rats showed significant increase in systolic blood pressure (F_(1,40)_ = 54.40, P<0.0001). Exposure to CRS induced significant (P<0.001) increase in systolic blood pressure in both non diabetic and diabetic rats in contrast to control groups. Chronic treatment with fluoxetine, but not imipramine significantly decreased blood pressure in both non diabetic (P<0.001) and diabetic rats (P<0.01) compared to vehicle treated group (F_(3,40)_ = 27.72, P<0.0001).

### Effect of tested drugs on insulin response in ITT

**Fig 4:**
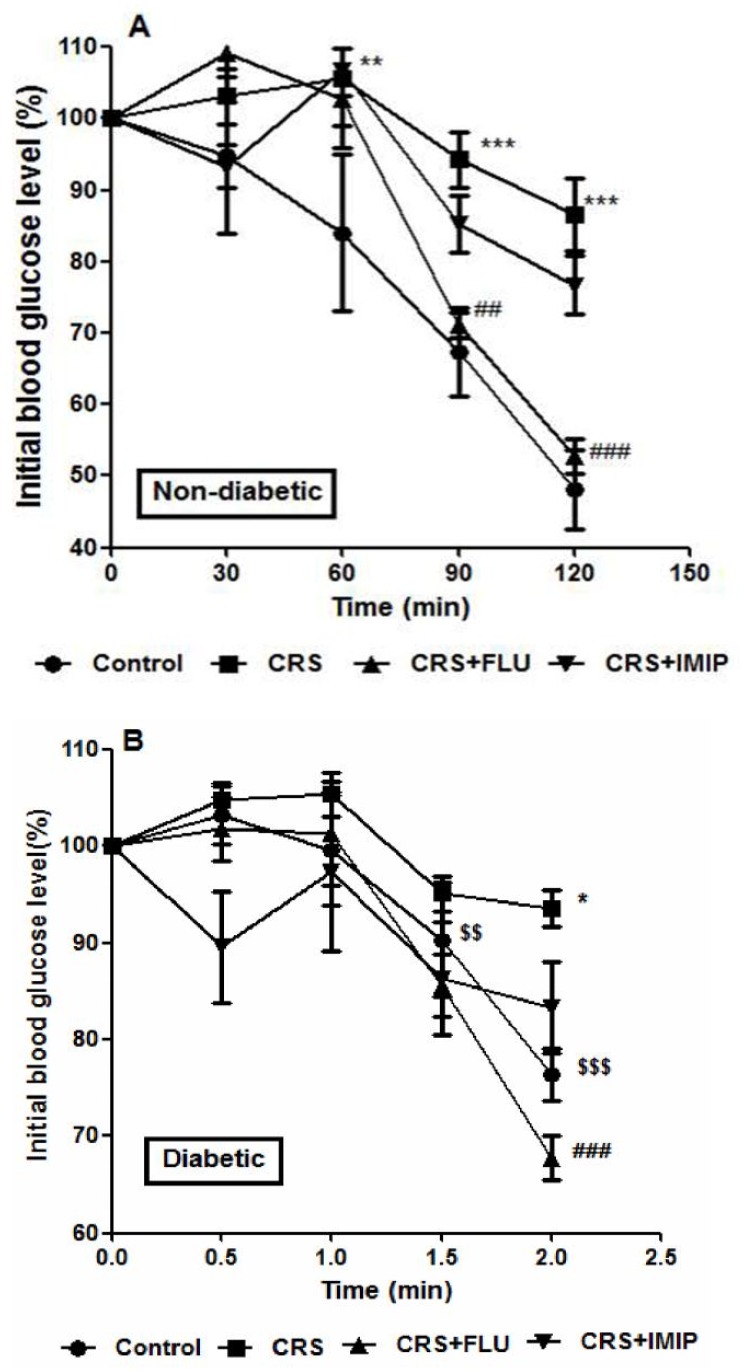
Effects of fluoxetine (FLU) and imipramine (IMIP) on percentage of initial blood glucose level during insulin tolerance test in non-diabetic (A) and diabetic (B) rats exposed to chronic restraint stress (CRS). Data are mean±SEM; (n = 6).*P<0.05, **P<0.01, ***P<0.001 vs. control group; ^##^P<0.01, ^###^P<0.001 vs. CRS group by Repeated-measures ANOVA with Bonferroni's post-hoc test. ^$$^P<0.01, ^$$$^P<0.001 diabetic vs. non-diabetic control rats.

Fig 4 shows that after insulin administration, the glucose concentration declined rapidly in control groups. Non-diabetic CRS rats showed significant (P<0.01, P<0.001, P<0.001) reduction in insulin response at 60, 90 and 120 min point measure respectively while diabetic CRS rats showed significant (P<0.05) reduction in insulin response at 120 min point measure, denoting the development insulin resistance. Fluoxetine, but not imipramine significantly (P<0.01, P<0.001) increased the insulin response in non-diabetic rats compared to vehicle-treated group at 90 and 120 min point measure while in diabetic rats fluoxetine produced significant (P<0.001) increase in insulin response at 120 min point measure compared to vehicle-treated group. Repeated measure ANOVA revealed significant effect of time (F_(4,120)_ = 59.855, P<0.0001), time with treatment (F_(8, 120)_ = 9.223, P<0.0001) and time with diabetes (F_(4,120)_ = 3.830, P = 0.006) factors.

### Effect of tested drugs on lipid profile and glucose homeostasis

Table 1 shows that induction of type 2 diabetes produced disturbance of lipid profile in Wistar rats manifested by significant increase in total cholesterol and triglycerides (F_(1,40)_ = 46.88, P<0.0001; F_(1,40)_ = 96.36, P<0.0001respectively). Exposure to CRS in non-diabetic and diabetic rats significantly increased total cholesterol (P<0.001; P<0.001),triglycerides (P<0.01; P<0.01) and LDL (P<0.001; P<0.01) and decreased HDL levels (P<0.001; P<0.01) respectively compared to control groups. Two-way ANOVA revealed that fluoxetine significantly decreased total cholesterol, triglycerides and LDL levels in non- diabetic and diabetic rats (F_(3,40)_ = 51.59, P<0.0001; F_(3,40)_ = 22.41, P<0.0001; F_(3,40)_ = 26.97, P<0.0001respectively) compared to vehicle-treated groups, while chronic treatment with imipramine exaggerated lipid profile disturbances in non diabetic rats; this was manifested by significant increase in total cholesterol and LDL levels (P<0.001; P<0.01respectively)compared to vehicle-treated groups.

**Table 1:** Effects of fluoxetine (FLU) and imipramine (IMIP) on lipid profile and glucose homeostasis in non-diabetic and diabetic rats exposed to chronic restraint stress (CRS).

Groups	Total cholesterol (mg/dl)	Triglycerides (mg/dl)	HDL (mg/dl)	LDL (mg/dl)	Fasting glucose (mg/dl)	Fasting insulin (μIU/ml)	HOMA-IR Index
Non-diabetic Rats							
Control	103.7±4.0	64.5±4.5	45.8±1.6	44.9±5.2	112.3±6.5	2.57±0.41	0.71±0.12
CRS	140.2±4.4***	114.7±7.3**	35.8±1.3***	81.4±6.2***	147.8±6.4*	16.43±1.16***	5.98±0.45***
CRS+FLU	114.3±2.8^##^	78.5±4.3^#^	39.0±4.1	59.6±4.0^#^	117.8±6.4^#^	11.60±0.70^##^	3.36±0.24^##^
CRS+IMIP	170.8±5.2^###^	132.5±7.6	34.3±0.7	110.3±5.9^##^	156.7±10.4	25.90±1.38^###^	9.98±0.72^###^
Diabetic Rats							
Control	142.7±6.0^$$$^	141.2±13.8^$$$^	43.7±0.7	70.8±7.1^$$^	170.3±2.3^$$$^	3.35±0.44	1.42±0.19
CRS	174.8±5.7***	186.7±16.5**	35.5±0.9**	102.0±7.1**	227.7±12.5***	15.35±0.65***	8.67±0.71***
CRS+FLU	131.0±6.0^###^	135.2±5.8^##^	41.0±0.4	63.0±6.7^###^	188.5±4.0^##^	7.82±0.68^###^	3.65±0.35^###^
CRS+IMIP	181.3±6.5	218.0±15.2	37.2±0.5	100.6±7.7	219.5±15.9	17.95±1.43	9.80±1.18

Data are mean±SEM (n = 6).

*P<0.05

**P<0.01

***P<0.001 vs control group;

^#^P<0.05

^##^P<0.01

^###^P<0.001 vs. CRS group by Two-way ANOVA with Bonferroni's post-hoc test.

^$$^P<0.01

^$$$^P<0.001 diabetic vs. non-diabetic control rats.

Furthermore, Table 1 indicated that diabetes produced disturbance of glucose homeostasis and insulin sensitivity manifested by significant elevation of fasting glucose, fasting insulin and HOMA IR index (F_(1,40)_ = 111.19, P<0.0001; F_(1,40)_ = 26.61, P<0.0001; F_(1,40)_ = 4.27, P<0.05 respectively). Similarly, CRS produced disturbance of glucose homeostasis manifested by significant increase in fasting glucose (P<0.05, P<0.001), fasting insulin (P<0.001; P<0.001) and HOMA IR index (P<0.001; P<0.001) in non-diabetic and diabetic rats respectively compared to control groups. Fluoxetine significantly reversed CRS-induced changes in glucose homeostasis (F_(3,40)_ = 13.89, P<0.0001; F_(3,40)_ = 151.41, P<0.0001; F_(3,40)_ = 86.53, P<0.0001 respectively), while imipramine exaggerated insulin resistance in non-diabetic rats as indicated by increased fasting insulin and HOMA IR index (P<0.001) compared to vehicle-treated groups.

### Effect of tested drugs on serum corticosterone

**Fig 5:**
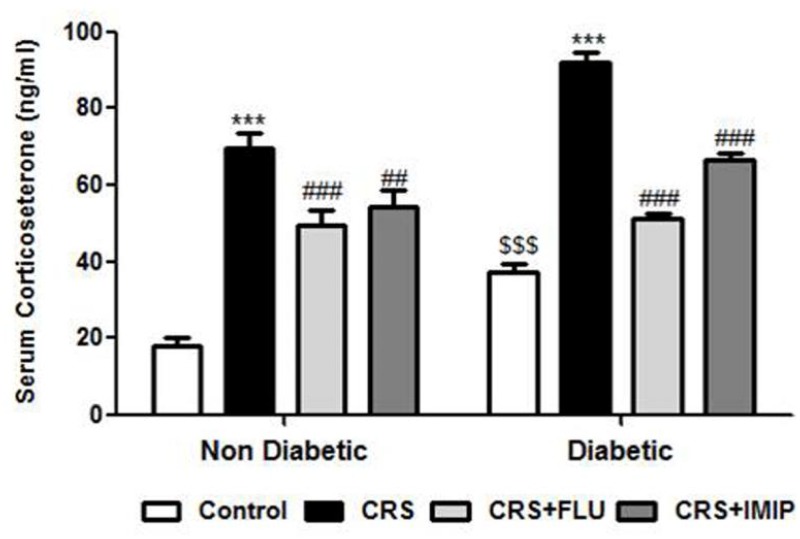
Effects of fluoxetine (FLU) versus imipramine (IMIP) on serum corticosterone in diabetic and non-diabetic rats exposed to chronic restraint stress (CRS). Data are mean±SEM (n = 6). ***P<0.001 vs. control group; ^##^P<0.01, ^###^P<0.001 vs. CRS group, ^$$$^P<0.001 diabetic vs. non-diabetic control rats by Two-way ANOVA with Bonferroni's post-hoc test.

As shown in Fig 5, diabetic rats showed significant activation of HPA axis indicated by increase in serum corticosterone (F_(1, 40)_ = 41.81, P<0.0001). Exposure to CRS induced significant (P<0.001) increase in serum corticosterone in both non diabetic and diabetic rats in contrast to control groups. Both fluoxetine and imipramine significantly decreased serum corticosterone in both non-diabetic (P<0.001 and P<0.01 respectively) and diabetic rats (P<0.001 and P<0.001) respectively compared to vehicle-treated group (F_(3, 40)_ = 106.36, P<0.0001).

### Effect of tested drugs on aortic TNF-α and IL-1ß proteins and TLR-4 gene and protein expression

As depicted in Table 2, exposure to diabetes induced significant increase in aortic TNF-α and IL-1ß (F_(1, 40)_ = 40.80, P<0.0001; F_(1, 40)_ = 69.11, P<0.0001 respectively) measured by ELISA. Exposure to CRS induced similar effects on TNF-α (P<0.05) and IL-1ß (P<0.01) in non-diabetic and diabetic rats. Chronic treatment with fluoxetine significantly decreased aortic TNF-α and IL-1ß proteins in both non diabetic (P<0.05) and diabetic rats (P<0.01 and P<0.05 respectively), while chronic treatment with imipramine significantly decreased IL-1ß in non-diabetic rats only with no significant effect on TNF-α either in non-diabetic or diabetic rats compared to vehicle treated group (F_(3,40)_ = 9.34, P<0.0001; F_(3, 40)_ = 8.08, P = 0.0003 for TNF-α and IL-1ß respectively).

**Table 2:** The effects of fluoxetine (FLU) versus imipramine (IMIP) on aortic TNF-α, IL-1ß and TLR-4 protein levels by ELISA technique in non-diabetic and diabetic rats exposed to chronic restraint stress (CRS).

Groups	Aortic TNF-*α* (ng/g protein)	Aortic IL-1*β* (ng/g protein)	Aortic TLR-4 (ng/g protein)
Non-diabetic Rats			
Control	33.1±3.75	6.10±1.04	2.42±0.46
CRS	64.6±7.38*	19.65±3.01**	13.00±2.71**
CRS+FLU	36.3±6.86^#^	8.86±1.37^#^	5.50±1.61^#^	
CRS+IMIP	55.9±11.79	9.79±1.01^#^	6.00±1.77^#^
Diabetic Rats			
Control	69.8±9.1^$$$^	22.93±1.50^$$$^	11.32±2.37^$$$^
CRS	102.1±7.0*	35.04±4.79**	18.83±1.49*
CRS+FLU	64.1±6.0^##^	25.15±3.78^#^	10.17±0.79^#^
CRS+IMIP	93.1±7.2	26.72±3.06	15.33±3.67

Data are mean±SEM (n = 6).

*P<0.05

**P<0.01 vs. control group;

^#^P<0.05

^##^P<0.01 vs. CRS group by Two-way ANOVA with Bonferroni's post-hoc test.

^$$^P<0.01

^$$$^P<0.001 diabetic vs. non-diabetic control rats.

**Fig 6:**
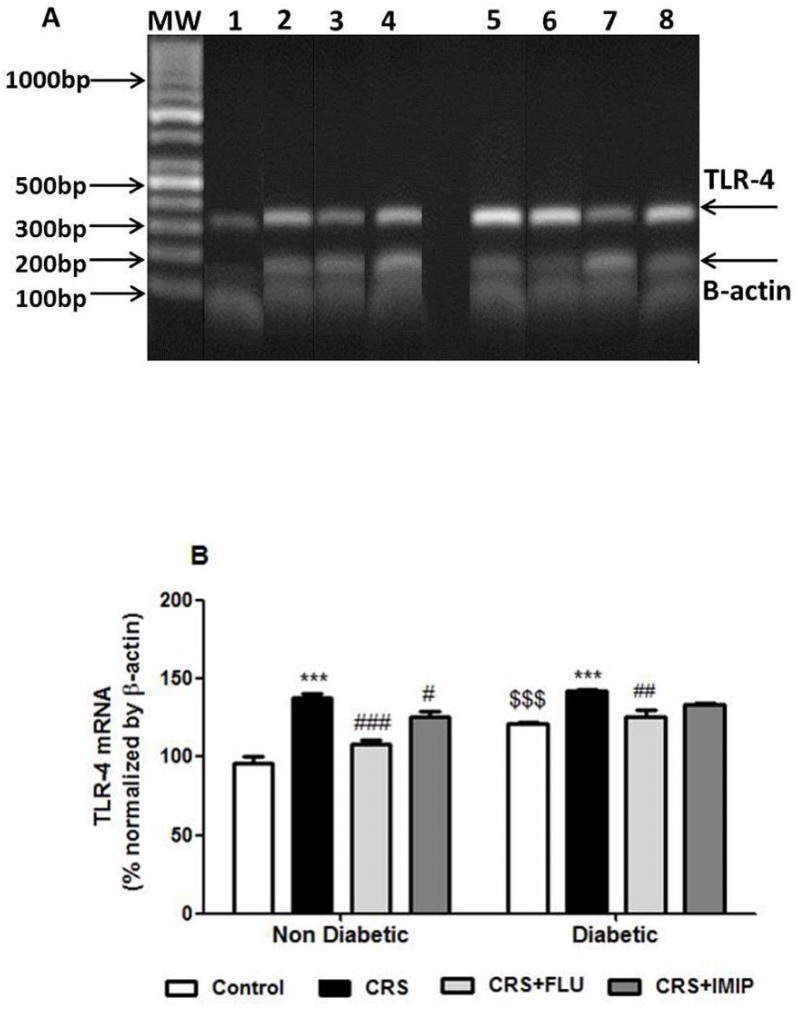
(A) Ethidium bromide-stained agarose gel electrophoresis showing the amplified RT-PCR products of TLR-4 (299bp) and β-actin (180 bp) as an internal standard, from aortic homogenates of Wistar rats. First Lane (MW): molecular weight ladder standard. Lane 1: control non-diabetic group. Lane 2: Non-diabetic/CRS vehicle-treated group. Lane 3: Non-diabetic/CRS fluoxetine-treated group. Lane 4: Non-diabetic/CRS imipramine-treated group. Lane 5: Control diabetic group. Lane 6: Diabetic/CRS vehicle-treated group. Lane 7: Diabetic/CRS fluoxetine-treated group. Lane 8: Diabetic/CRS imipramine-treated group. (B) Effects of fluoxetine (FLU) versus imipramine (IMIP) on aortic TLR-4 gene expression (mRNA) as % normalized by β-actin quantity using semi-quantitative RT-PCR in diabetic and non-diabetic rats exposed to chronic restraint stress (CRS). Data are mean±SEM (n = 6). ***P<0.001 vs. control group; ^#^P<0.05, ^##^P<0.01, ^###^P<0.001 vs. CRS group, ^$$$^P<0.001 diabetic vs. non-diabetic control rats by Two-way ANOVA with Bonferroni's post-hoc test.

Fig 6 and Table 2 reveal that, diabetic rats showed significant increase in aortic TLR-4 gene and protein expression (F_(1,40)_ = 43.09, P<0.0001 and F_(1,40)_ = 23.46, P<0.0001 respectively). Exposure to CRS induced significant increase in TLR-4 gene expression and protein expression in non-diabetic (P<0.001, P<0.01) and diabetic (P<0.001, P<0.05) rats respectively in contrast to control groups. Chronic treatment with fluoxetine could significantly decrease TLR-4 gene and protein expression in both non diabetic (P<0.001, P<0.05) and diabetic rats (P<0.01, P<0.05) respectively while imipramine significant decreased TLR-4 gene and protein expression in non-diabetic rats (P<0.05) only as compared to vehicle treated group (F_(3,40)_ = 44.19, P<0.0001; F_(3,40)_ = 7.51, P = 0.0004 respectively).

### Effect of tested drugs on rat’s aortic ring vascular reactivity and histopathological changes

**Fig 7:**
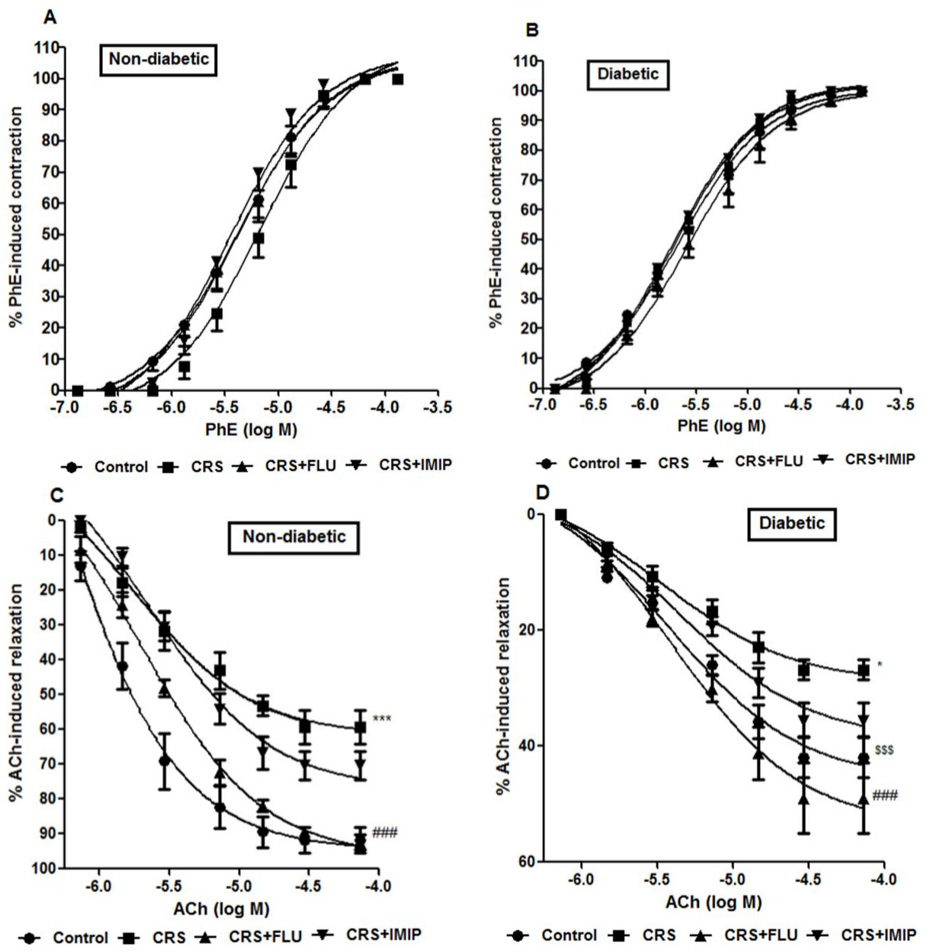
Effects of fluoxetine (FLU) versus imipramine (IMIP) on % phenylephrine (PhE)-induced contraction in non-diabetic (A) & diabetic rats (B) and maximal relaxation response (%) of acetylcholine in non-diabetic (C) & diabetic rats (D) exposed to chronic restraint stress (CRS). Data are mean±SEM; (n = 6). *P<0.05, ***P<0.001 vs. control group; ^###^P<0.001 vs. CRS group by Two-way ANOVA with Bonferroni's post-hoc test.^$$$^P<0.001 diabetic vs. non-diabetic control rats.

Fig 7 and Table 3 indicate that diabetes induced significant increase in vascular reactivity to. vasoconstrictors [manifested by increase in phenylephrine Emax (P<0.001)] and decline in endothelium-dependent vascular relaxation of PhE-precontracted aortic ring in response to acetylcholine (P<0.001) compared to non-diabetic control rats (F_(1, 40)_ = 674.95, P<0.0001; F_(1,40)_ = 216.08, P<0.0001respectively). Exposure to CRS significantly decreased endothelium-mediated vascular relaxation (P<0.001; P<0.05) in non-diabetic and diabetic rats respectively compared to control groups. Two-way ANOVA revealed that chronic treatment with fluoxetine but not imipramine significantly (P<0.001) reversed CRS induced decrease in endothelium-mediated vascular relaxation in non-diabetic and diabetic rats compared to vehicle-treated groups (F_(3, 40)_ = 21.66, P<0.0001). Statistical analysis revealed no differences in SNP-induced maximal relaxation response among groups indicating that endothelium-independent relaxation was not affected in the present experiment.

**Table 3:** Effects of fluoxetine (FLU) versus imipramine (IMIP) on isolated aortic ring phenylephrine (PhE) contractile response (EC_50 and_ Emax) and maximal relaxation response (%) of acetylcholine (Ach) and sodium nitroprusside (SNP) in non-diabetic and diabetic rats exposed to chronic restraint stress (CRS).

Groups	PhE EC_50_ (μM)	PhE Emax (g tension)	Ach Maximal Relaxation (%)	SNP Maximal Relaxation (%)
Non-diabetic Rats				
Control	3.33±0.72	0.52±0.02	91.8±3.5	98.3±0.7
CRS	4.21±1.24	0.48±0.01	59.4±4.9***	96.0±1.5
CRS+FLU	4.99±1.20	0.50±0.03	92.3±2.2^###^	96.8±1.0
CRS+IMIP	3.71±0.57	0.47±0.03	70.5±4.1	95.7±1.1
Diabetic Rats				
Control	4.19±1.36	1.20± 0.03^$$$^	42.0±3.5^$$$^	95.5±1.9
CRS	3.38±1.14	1.19± 0.06	26.9±1.7*	95.5±1.4
CRS+FLU	3.06±0.64	1.18± 0.04	49.0±6.1^###^	96.5±1.8
CRS+IMIP	3.23±1.18	1.30± 0.06	35.6±3.0	96.0±1.9

Data are mean±SEM (n = 6).

*P<0.05

***P<0.001vs control group;

^###^P<0.001 vs. CRS group by Two-way ANOVA with Bonferroni's post-hoc test.

^$$$^P<0.001 diabetic vs. non-diabetic control rats.

**Fig 8:**
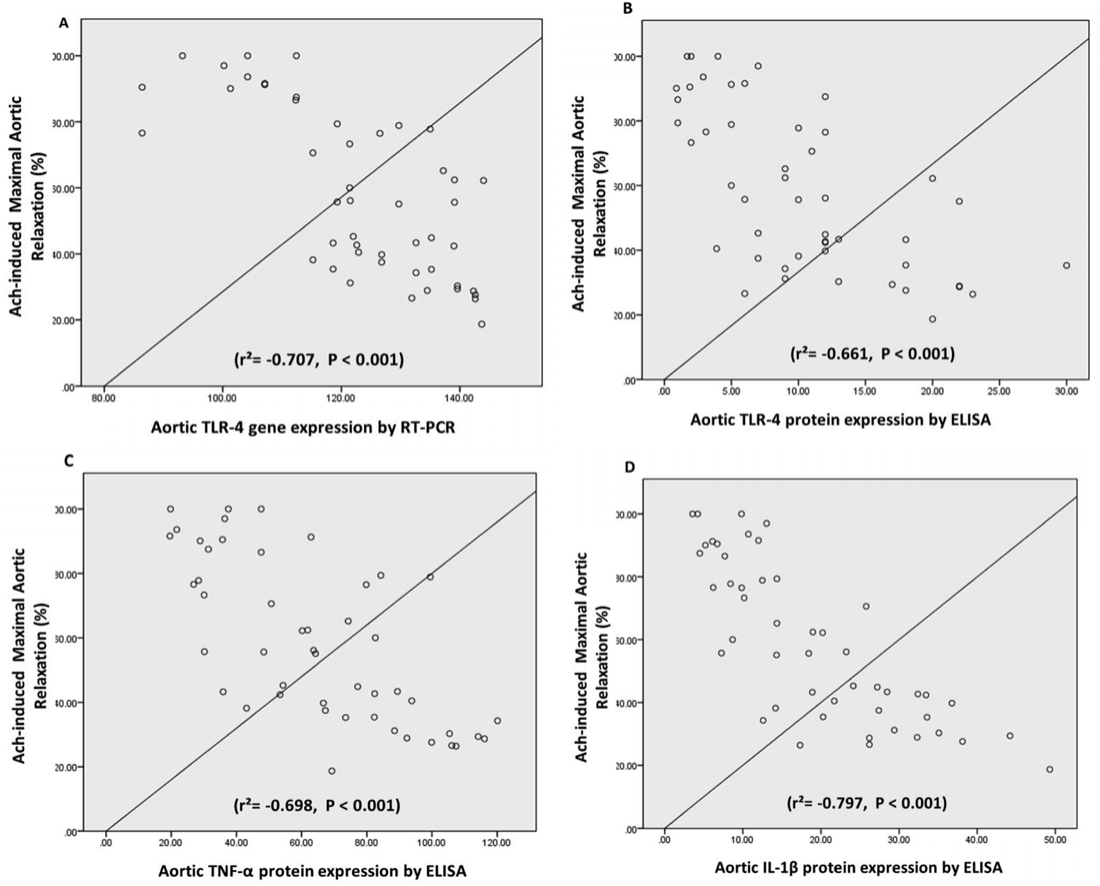
Pearson's correlation between Ach-induced maximal aortic relaxation response (%) and aortic expression of inflammatory markers, TLR-4 gene (mRNA by RT-PCR), TLR-4 protein, TNF-α and IL-1β at the present study.

As shown in Fig 8, statistical analysis by Pearson’s correlation coefficient reveals that Ach-induced maximal aortic relaxation response (%) significantly correlates with aortic expression of inflammatory markers, TLR-4 gene (r^2^ = -0.707, P < 0.001), TLR-4 protein (r^2^ = -0.661, P < 0.001), TNF-α (r^2^ = -0.698, P < 0.001) and IL-1β (r^2^ = -0.797, P < 0.001).

**Fig 9:**
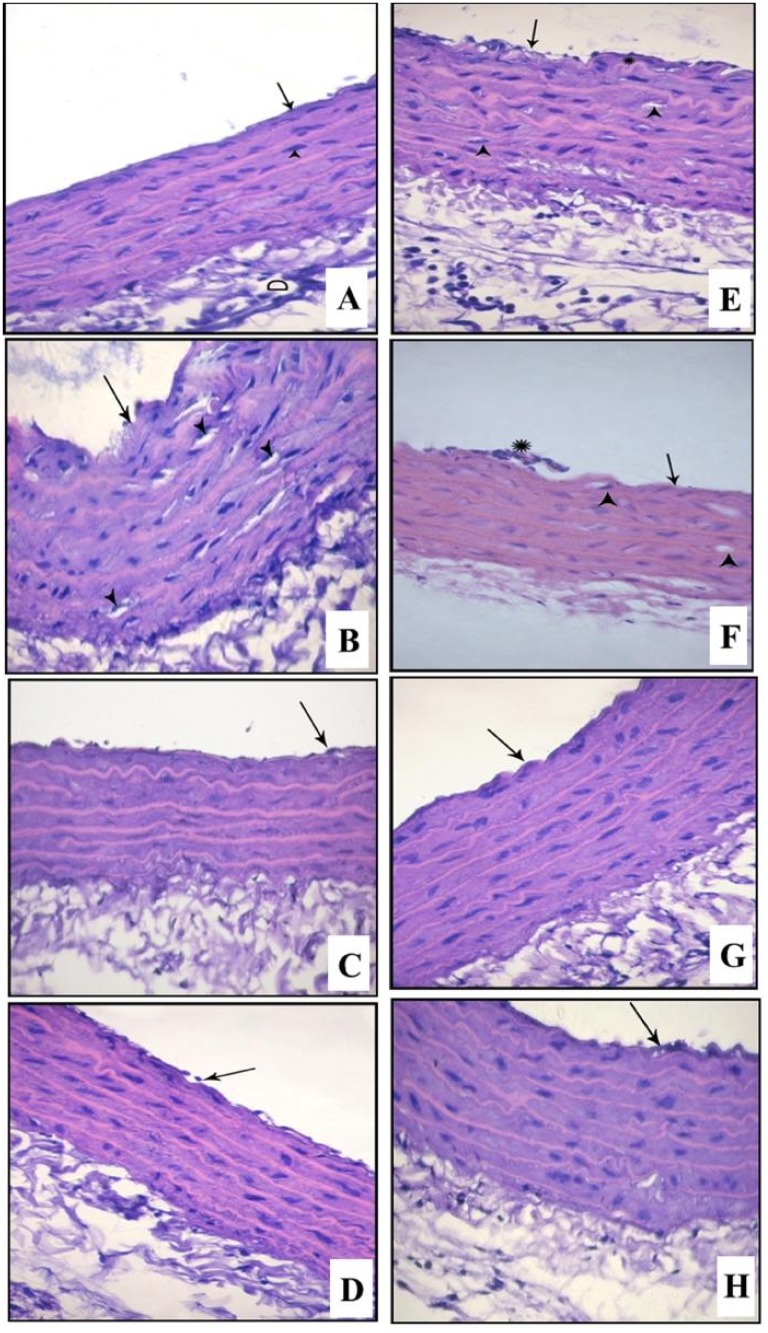
Photomicrographs of aortic sections stained by H&E (X640) in different experimental groups. (A) Control non-diabetic group with unremarkable change, groups (B) Non-diabetic/CRS vehicle-treated group, (C) Non-diabetic/CRS fluoxetine-treated group (D) Non-diabetic/CRS imipramine-treated group, (E) Control diabetic group (F) Diabetic/CRS vehicle-treated group, (G) Diabetic/CRS fluoxetine-treated group, (H) Diabetic/CRS imipramine-treated group. Endothelial lining of tunica intima (↑) and vacuolation of cytoplasm of some smooth muscle fibers (▲).

Microscopic examination (Fig 9 and Table 4) of H&E stained sections obtained from CRS vehicle-treated group showed discontinuation of the endothelial layer of the tunica intima, splitting of the elastic fibers of the media and vacuolation of cytoplasm of some smooth muscle fibers with significant increase in intima/media ratio (P<0.01). CRS fluoxetine-treated group showed that the structure of aorta was more or less comparable to control group indicating recovery of histopathological changes induced by CRS, while CRS imipramine-treated group showed focal discontinuity of endothelial lining of tunica intima. Sections from control diabetic group showed discontinuation of the endothelial layer of the tunica intima, splitting of the elastic fibers of the media and vacuolation of cytoplasm of some smooth muscle fibers with significant increase in intima/media ratio (P<0.001). Sections from thoracic aorta of diabetes/CRS vehicle-treated group showed exaggeration of these changes. Sections from diabetic CRS fluoxetine-treated group showed significant amelioration of intima/media ratio (P<0.001) with focal loss of endothelial lining of tunica intima with branching of few elastic lamella. Sections from diabetic/CRS imipramine- treated group showed insignificant reduction in intima/media ratio compared to vehicle treated group.

**Table 4:** The effects of fluoxetine (FLU) versus imipramine (IMIP) on aortic intima/media ratio (H & E staining) and immunohistochemical staining of aortic TNF-α (optical density) in non-diabetic and diabetic rats exposed to chronic restraint stress (CRS).

Groups	Aortic H & E staining Intima /media ratio X100	Immunohistochemical staining Aortic TNF-*α* optical density
Non-diabetic Rats		
Control	0.90±0.05	0.614 ± 0.023
CRS	1.39±0.08**	0.891 ± 0.015**
CRS+FLU	1.05±0.09	0.701 ± 0.008^#^
CRS+IMIP	1.54±0.10	0.788 ± 0.001
Diabetic Rats		
Control	1.66±0.10^$$$^	0.822 ± 0.041^$$^
CRS	3.64±0.20***	0.993 ± 0.065*
CRS+FLU	1.76±0.06^###^	0.804 ± 0.005^#^
CRS+IMIP	3.34±0.16	0.811 ± 0.074

Data are mean±SEM (n = 6).

*P<0.05

**P<0.01

***P<0.001 vs. control group;

^#^P<0.05

^###^P<0.001 vs. CRS group by Two-way ANOVA with Bonferroni's post-hoc test.

^$$^P<0.01

^$$$^P<0.001 diabetic vs. non-diabetic control rats.

**Fig 10:**
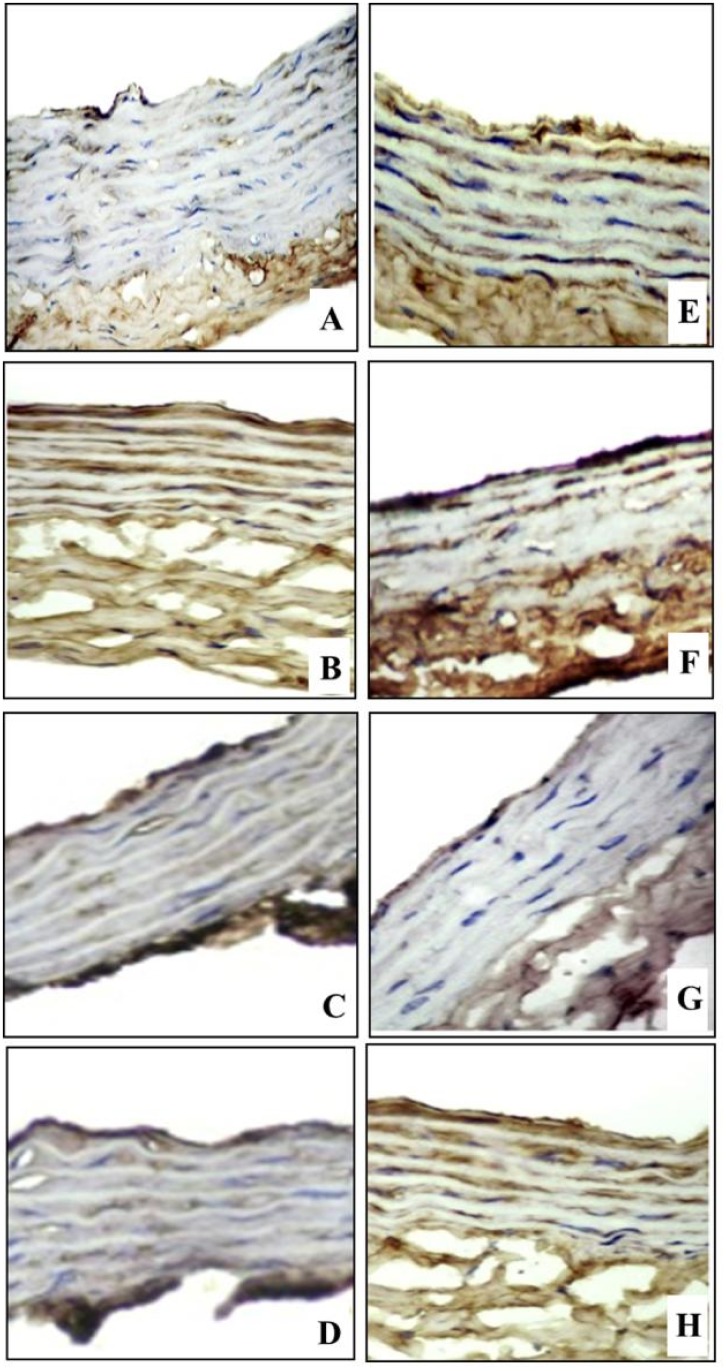
TNF-α immunohistochemical staining (IHCX400) of aortic sections in different experimental groups. (A) Control non-diabetic group with focal faint TNF-a immunostaining (brownish color), (B) Non-diabetic/CRS vehicle-treated group shows moderate diffuse immunostaining, (C) Non-diabetic/CRS fluoxetine-treated and (D) Non-diabetic/CRS imipramine-treated groups show focal mild staining, (E) Control diabetic group (F) Diabetic/CRS vehicle-treated group shows strong diffuse immunostaining, (G) Diabetic/CRS fluoxetine-treated group shows focal mild staining and (H) Diabetic/CRS imipramine-treated group show moderate diffuse immunostaining.

As depicted in Fig 10 and Table 4, exposure to diabetes induced significant increase in aortic immune-staining optical density of TNF-α (F_(1, 40)_ = 10.23, P = 0.0024). Exposure to CRS induced similar effects on immune-staining optical density of TNF-α in non-diabetic and diabetic rats (P<0.01, P<0.05 respectively). Chronic treatment with fluoxetine significantly (F_(3,40)_ = 12.14, P = 0.0051) decreased aortic TNF-α immune-staining in both non diabetic (P<0.05) and diabetic rats (P<0.05), while chronic treatment with imipramine insignificantly affected TNF-α either in non-diabetic or diabetic rats compared to vehicle treated group.

## Discussion

In the present work, exposure to CRS as well as DM induced depressive-like behavior manifested by increased immobility time in FST, decreased body weight gain and increased serum corticosterone levels. Chronic treatment with FLU and IMIP reversed these changes in a comparable manner. This is consistent with recent studies which reported CRS-induced depressive-like behavior manifested by increased immobility time in FST, decreased body weight and increased serum corticosterone levels in Wistar rats [17, 18, 28]. Previous animal studies have shown that diabetic mice and rats presented higher depressive-like behavior when submitted to FST [29, 30]. Wang et al. [31] suggested that diabetes-induced reduction of neurogenesis in hippocampus implies a potential mechanism for diabetes-related depression and cognitive decline. Hyperactivity of HPA axis is well documented event in depression [32] and the dysregulation of the HPA axis has been regarded as a neuro-endocrine hallmark of chronic stress, at the same time, HPA axis dysfunction is a possible pathological element in DM. Previous reports showed that plasma ACTH and corticosterone levels are higher in uncontrolled diabetic rats [33]. Literature data show that fluoxetine and imipramine dose-dependently reversed behavioral changes mediated by exposure to diabetes and psychological stress [29, 34]. Fluoxetine and imipramine increase biogenic amines and hippocampal BDNF and correct the HPA dysfunction as indicated by the decline of serum corticosterone level [35]. The ability of fluoxetine to reverse stress induced behavioral changes in our diabetic high fat diet (HFD) fed rats is in contrast to Isingrini et al. [36] who reported HFD-induced fluoxetine resistance and this inconsistency may be derived from changes in animals used, composition of HFD, duration and type of stress. They used mice exposed to unpredictable chronic mild stress for 14 weeks in contrast to Wister rats exposed to CRS for 6 weeks used in our study.

In the present work, DM and CRS significantly increased serum lipids, glucose and HOMA-IR index with defective insulin response in ITT. This was accompanied by aortic atherosclerotic changes (increase in aortic intima/media ratio, impaired endothelial-dependent relaxation and elevated SBP). Prolonged hyperglycemia is a major factor in the pathogenesis of atherosclerosis with diabetes. Non-enzymatic glycosylation of proteins and lipids can interfere with their normal function; in addition, glycosylated proteins interact with a specific receptor present on all cells including monocyte-derived macrophages, endothelial cells, and smooth muscle cells resulting in the induction of oxidative stress and pro-inflammatory responses [37]. Additionally, the degree of insulin resistance relates directly to increasing rates of myocardial infarction and stroke [38]. Diabetic dyslipidemia may be another risk factor for subsequent cardiovascular diseases. Previous studies reported disturbance of lipid profile, decreased body weight gain, development of atherosclerotic changes with increased intima/media and impaired endothelium-dependent relaxation in STZ-induced diabetic rats [16].

Under chronic stress conditions, elevated corticosteroids may cause insulin resistance which facilitates TG synthesis in the liver and may delay the clearance of lipoproteins, also resulting in hypercholesterolemia, decrease binding and degradation of LDL by liver cells [39]. Chronic stress induced pro-atherogenic changes, elevated blood pressure, insulin resistance with increased super-sensitivity to Phenylephrine in aortic rings with lower relaxation response to acetylcholine, this effect seems to be related to decrease in the bioavailability of endothelial NO induced by stress [40].

The present work emphasizes the improvement of metabolic and vascular effects induced by DM and CRS with chronic fluoxetine treatment in contrast to no improvement or even worsening of these effects with imipramine. Several previous studies have reported the favorable effect of chronic fluoxetine treatment on blood glucose and lipids [41, 42]; this effect may be due to improvement of insulin sensitivity which is noticed in this work, fluoxetine also have an antioxidant effect [43]. Breum et al. [44] reported that chronic fluoxetine treatment improved glycemic control in patients with NIDDM with improvement of insulin sensitivity. The suppression of the hyperactive HPA axis with amelioration of stress may also have an impact on improvement of insulin resistance. Isingrini et al. [45] reported improvement of endothelial dysfunction and SBP induced by chronic stress back to normal with chronic fluoxetine treatment. Also, Amsterdam et al. [46] observed reduction in blood pressure with fluoxetine in patients with major depressive disorders.

The serotonin (5-HT) transporter is present and functional in endothelial and arterial smooth muscle cells. By inhibiting this transporter, fluoxetine can increase the 5-HT level in the microenvironment, and 5-HT can stimulate 5HT2B endothelial receptors [47], eliciting a release of NO and the subsequent elevation of cyclic guanosine monophosphate leading to relaxation in the underlying smooth muscle cell [48]. This effect would be dependent on endothelial NO synthase activation by phosphorylation [49]. This hypothesis is supported by the fact that SSRI treatment in depressed patients induces both an increase of the plasma 5-HT level and an increase of the plasma NO level [50].

In contrast, imipramine did not improve DM-CRS induced metabolic and vascular effects, but it aggravated the disturbance of glycemic control and lipid profile. Regarding the mechanisms of imipramine-induced glucose deregulation, it has been reported that imipramine inhibits the synaptic reuptake of nor-epinephrine and serotonin (5-HT) at nerve terminals. Nor-epinephrine may stimulate glycogenolysis and gluconeogenesis resulting in raised blood glucose levels and subsequent insulin resistance [51]. Moreover, Ghaeli et al. [41] and Salehi and Sanjani [52] revealed increase in fasting blood glucose upon chronic treatment with imipramine. Ananloo et al. [42] reported increased serum total cholesterol, triglyceride and body weight in depressive patients treated with imipramine. Indeed, previous reports revealed imipramine-induced dyslipidemia, insulin resistance and endothelial dysfunction in CMS model [53], but this is the first study showing this observation in CRS paradigm.

Our results demonstrated a significant increase in aortic TLR-4 gene and protein expression parallel to a similar increase in aortic levels of the pro-inflammatory cytokines TNF-α and IL1-β in diabetic and stressed rats. Moreover, Pearson’s correlation coefficient revealed that endothelial dysfunction significantly correlated with aortic expression of inflammatory markers, TLR-4, TNF-α and IL-1β. Indeed, chronic inflammation is a common factor in insulin resistance and type 2 DM. Consistently, Dasu et al. [54] have shown increased TLR-4 expression, intracellular signaling and TLR-mediated inflammation in monocytes with a significant correlation with HbA1c levels in diabetic patients. Kuwabara et al. [55] reported increased TLR-4 expression in diabetic patients with diabetic nephropathy while, Reyna et al. [56] demonstrated increased muscle TLR4-driven signaling in insulin-resistant subjects.

In endothelial cells, activation of TLR-4 promotes elevated reactive oxidative stress, reduces eNOS coupling leading to a reduced NO-production and bioavailability leading to endothelial dysfunction [57]. Moreover, antagonizing TLR is effective in reducing vascular inflammation and early stage atherosclerosis in diabetic mice [12].

Hyperglycemia markedly increased TLR-4 mRNA and protein expression in macrophages of atherosclerotic lesions [58] and increased levels of NF-κB that promoted inflammatory cytokines (TNF-α, IL-1ß, and adhesion molecules) leading to atherosclerosis [59]. Furthermore, increased oxLDL and free fatty acids levels associated with DM could stimulate TLR-4 gene expression and protein content [56, 60].

TLR-4 expression with TNF-α and IL-1β production are increased in atherosclerotic plaques and surrounding tissues [61]. Previous studies reported that chronic psychological stress could stimulate TLR-4 signaling pathway in various tissues including blood vessels with significant correlation to atherosclerosis [14]. Clinical and experimental studies showed that corticosteroids, TNF-α and IL-1 ß serum level were significantly higher in individuals with psychological symptoms [62] and animals treated with chronic stress [63].

Chronic treatment with fluoxetine induced significant reduction in aortic TLR-4 expression with reduction in TNF-α and IL-1ß level, these effects are parallel to amelioration of vascular dysfunction in diabetic and non-diabetic rats. While, imipramine reduced aortic TLR-4 expression and IL-1ß levels in non-diabetic rats with no significant effect on TNF-α.

Sacre et al. [64] elucidated that fluoxetine reduced TLR expression with subsequent reduction in cytokine level (IL6, TNF-α and IFN-γ) in murine model of rheumatoid arthritis. As for imipramine, was able to reverse the alterations on TNF-α and IL-1ß in serum and CSF of rats submitted to animal model of maternal deprivation [65]. At the same time, imipramine was reported to enhance the production of the negative immune-regulator IL-10 in rats subjected to FST induced stress [66]. In a recent study, imipramine treatment induced a decline in the gene expression of TNF-α induced by LPS/CMS protocol [67].

The preferable anti-inflammatory effect of fluoxetine over imipramine was discussed in a study by Roumestan et al. [68]; they noticed preferable effect of fluoxetine over desipramine, metabolite of imipramine, by the evident sensitivity of IL-1ß to the suppressive effect of imipramine than TNF-α. Another study found that following a LPS challenge in rats, TNF-α was more sensitive to a higher dose of imipramine than IL-1ß [69]. The mechanism by which drugs can differentially modulate IL-1ß and TNF-α is presently unknown.

In conclusion, the present work implies that a chronic inflammatory state underlies diabetes-psychological stress associated vascular effects with rising role of TLR-4 and subsequent cytokine production. To our knowledge, our study is the first to elucidate the preferable anti-inflammatory effect of fluoxetine over imipramine as regards reducing gene expression of TLR-4 in atherosclerotic aortic lesions in diabetic/stressed subjects. Since fluoxetine was also associated with preferable effect on glucose homeostasis and lipid profile which are markedly disturbed in diabetes, this work clarifies the preference of fluoxetine over imipramine in management of depression with type 2DM.
